# Inhibition of PRMT5 Attenuates Oxidative Stress-Induced Pyroptosis via Activation of the Nrf2/HO-1 Signal Pathway in a Mouse Model of Renal Ischemia-Reperfusion Injury

**DOI:** 10.1155/2019/2345658

**Published:** 2019-11-25

**Authors:** Changhui Diao, Zhiyuan Chen, Tao Qiu, Hao Liu, Yuanyuan Yang, Xiuheng Liu, Junfeng Wu, Lei Wang

**Affiliations:** ^1^Department of Urology, Renmin Hospital of Wuhan University, Wuhan, Hubei, China; ^2^Department of Organ Transplantation, Renmin Hospital of Wuhan University, Wuhan, Hubei, China

## Abstract

**Background:**

Extensive evidence has demonstrated that oxidative stress, pyroptosis, and proinflammatory programmed cell death are related to renal ischemia/reperfusion (I/R) injury. However, the underlying mechanism remains to be illustrated. Protein arginine methylation transferase 5 (PRMT5), which mediates arginine methylation involved in the regulation of epigenetics, exhibits a variety of biological functions and essential roles in diseases. The present study investigated the role of PRMT5 in oxidative stress and pyroptosis induced by I/R injury in a mouse model and in a hypoxia/reoxygenation (H/R) model of HK-2 cells.

**Methods:**

C57 mice were used as an animal model. All mice underwent right nephrectomy, and the left renal pedicles were either clamped or not. Renal I/R injury was induced by ligating the left renal pedicle for 30 min followed by reperfusion for 24 h. HK-2 cells were exposed to normal conditions or stimulation through H/R. EPZ015666(EPZ)—a selective potent chemical inhibitor—and small interfering RNA (siRNA) were administered to suppress the function and expression of PRMT5. The levels of urea nitrogen and creatinine in the serum and renal tissue injury were assessed. Immunohistochemistry, western blotting, and reverse transcription-polymerase chain reaction were used to evaluate pyroptosis-related proteins including nod-like receptor protein-3, ASC, caspase-1, caspase-11, GSDMD-N, and interleukin-1*β*. Cell apoptosis and cell viability were detected through flow cytometry, and the levels of reactive oxygen species (ROS) and hydrogen peroxide (H_2_O_2_) were measured. Ki-67 was used to assess the proliferation of renal tubular epithelium. In addition, the activity of malondialdehyde and superoxide dismutase was determined.

**Results:**

I/R or H/R induced an increase in the expression of PRMT5. Inhibition of PRMT5 by EPZ alleviated oxidative stress and I/R- or H/R-induced pyroptosis. In renal tissue, the application of EPZ promoted the proliferation of tubular epithelium. In addition, H/R-induced pyroptosis in HK-2 cells was dependent on oxidative stress in vitro. Administration of either EPZ or siRNA led to decreased expression of pyroptosis-related proteins. Inhibition of PRMT5 also attenuated the I/R- or H/R-induced oxidative stress in vivo and in HK-2 cells, respectively. It also resulted in a distinct decrease in the levels of malondialdehyde and H_2_O_2_, and an apparent increase in superoxide dismutase activity in mouse renal tissue. Moreover, it led to a significant decrease in the levels of ROS and H_2_O_2_ in HK-2 cells. When activated, NF-E2-related factor/heme oxygenase-1 (Nrf2/HO-1)—a key regulator of various cytoprotective proteins that withstand oxidative damage—can decrease the generation of ROS. Nrf2/HO-1 was downregulated during I/R in tissues and H/R in HK-2 cells, and this effect was reversed by the PRMT5 inhibitor. Furthermore, the expressions of Nrf2 and HO-1 proteins were markedly upregulated by EPZ or siRNA against PRMT5.

**Conclusion:**

PRMT5 is involved in ischemia- and hypoxia-induced oxidative stress and pyroptosis in vitro and in vivo. Inhibition of PRMT5 may ameliorate renal I/R injury by suppressing oxidative stress and pyroptosis via the activation of the Nrf2/HO-1 pathway, as well as promoting the proliferation of tubular epithelium. Therefore, PRMT5 may be a promising therapeutic target.

## 1. Introduction

Acute kidney injury (AKI) is a clinical complication that often occurs after kidney transplantation, severe trauma, partial nephrectomy, nephrotoxicity, renal artery thromboembolism, sepsis, etc. The most common cause of AKI is ischemia and reperfusion (I/R) injury [1–3]. AKI often results in the accumulation of metabolic waste, water and electrolyte disorders, and a series of clinical features including edema, hypertension, and hemorrhage [4, 5].

The protein arginine methyltransferase (PRMT) family catalyze arginine monomethylation and asymmetric or symmetric dimethylation modifications which are conserved and ubiquitous physiological processes. PRMTs play key roles in multiple biological processes [6]. There are two types of PRMTs: type I (i.e., PRMT 1, 3, and 4) catalyzing the conversion of arginine to a monomethylarginine intermediate, followed by conversion of asymmetric dimethylarginine and type II (i.e., PRMT5) catalyzing the formation of symmetric dimethylarginine [7, 8]. Previous studies reported that hypoxia- and ischemia-induced apoptosis in human lung epithelial cells in pigs was regulated by the activation of PRMT1 and PRMT5 [9] and that the expression of PRMT5 was increased in the renal tubular epithelium of the animal model of ischemic/reperfusion injury [10]. However, the role of PRMT5 in oxidative stress and inflammation induced by renal I/R has not been elucidated.

Increasing evidence indicated that the generation of reactive oxygen species (ROS) and the inflammatory response affected the severity of renal I/R injury [11–13]. Ischemia-induced dysfunctional mitochondria lead to an abundant production of ROS, followed by the activation of inflammasomes, which are mediators of inflammatory responses. Simultaneously, activation of inflammasomes can be repressed by antioxidants, such as N-acetyl-L-cysteine (NAC) [14, 15], silent information regulator 1 [16], and numerous bioactive substances [17–19]. Inflammation plays a crucial role in tissue repair, which is a response to damage [20]. Inflammasomes, often activated by pathogen- and damage-associated molecular patterns, mediate the production of proinflammatory cytokines, such as mature interleukin-1*β* (IL-1*β*) [21]. At present, the most well-characterized inflammasome is the nod-like receptor protein-3 (NLRP3) inflammasome, which is composed of NLRP3, ASC, and procaspase-1. The NLRP3 inflammasome catalyzes the conversion of procaspase-1 to activated caspase-1, subsequently leading to the maturation and secretion of IL-1*β* [22]. The NLRP3 inflammasome, containing caspase-1, is a multiprotein complex that controls the maturation and release of IL-1*β* and plays a key role in pyroptosis [23]. Caspase-11 and Gasdermin D (GSDMD) were also classical pyroptotic markers [24]. Caspase-11, a cysteine protease, can activate NLRP3 and GSDMD to promote cell pyroptosis. GSDMD, a specific substrate of caspase-11 and caspase-1, can be cleaved to generate an amino terminal GSDMD-N and a carboxyl terminal GSDMD-C, and GSDMD-N, an active pore-forming protein, promotes leakage of inflammasome such as IL-1*β*, disrupt the osmotic potential, and eventually induce cell pyroptosis [24, 25].

Pyroptosis is a type of distinct cell death, which is distinct from apoptosis and necrosis. It is a general and natural immune effector mechanism, contributing to the inflammatory reaction in bacterial infections and various noninfectious diseases [26–28]. The activation of caspase-1 and inflammasomes is responsible for pyroptosis [29, 30]. Studies have shown that pyroptosis is involved in I/R injury of the lungs and heart [31, 32]. Therefore, we hypothesized that in a renal I/R model, ROS-mediated activation of the NLRP3 inflammasome and pyroptosis play a vital role in renal damage. Furthermore, the present study investigated the relationship between PRMT5 and ROS, as well as potential mechanisms involved in this process.

## 2. Materials and Methods

### 2.1. Antibodies and Reagents

EPZ015666 (GSK 3235025) (EPZ) was purchased from Selleck Chemicals (Houston, TX, USA). NAC and polyethylene glycol-catalase were supplied by Sigma–Aldrich (St. Louis, MO, USA). Antibodies used in western blotting (WB) and immunohistochemistry were as follows. The rabbit anti-PRMT5 and anti-GAPDH were purchased from Santa Cruz Biotechnology (Santa Cruz, CA, USA). Antibodies against NLRP3, ASC, and caspase-1 and caspase-11 and GSDMD-N were obtained from Santa Cruz Biotechnology (Santa Cruz, CA, USA), while antibodies against Nrf2, HO-1, IL-1*β*, and Ki-67 were purchased from Abcam (Cambridge, UK). Dichlorofluorescein diacetate (DCFH-DA) solution was supplied by Beyotime Biotechnology (Jiangsu, China). Creatinine and urea commercial kits, the superoxide dismutase (SOD) assay kit, and the malondialdehyde (MDA) assay kit were purchased from Nanjing Jiancheng Bioengineering Institute (Nanjing, China).

### 2.2. Experimental Animals and the I/R Model

Adult male C57BL6 (C57) mice, weighing 20–25 g (age: 8–12 months), were supplied by the First Clinical College Experimental Animal Center of Wuhan University, Wuhan, China, and housed under a temperature of 20–22°C with a 12-h light/dark cycle. The mice had ad libitum access to water and standard chow. All procedures were performed in accordance with the guidelines for the care and use of laboratory animals published by the National Institutes of Health. EPZ was solute with dimethyl sulfoxide and injected intraperitoneally once a day for 1 week in the EPZ-treated groups prior to establishing the model. Different dosages were used according to the experimental design. The I/R model was performed as follows. Briefly, an intraperitoneal injection of pentobarbital (50 mg/kg) was adopted to anesthetize the animals. Subsequently, the mice were placed on a thermostatic surgical plate to maintain a core temperature of 37°C. During surgery, a midline incision was performed and the right kidney was excised. The left renal pedicles were clamped for 30 min. However, these pedicles were not clamped in the sham group. According to the experimental design, the mice were euthanized and sacrificed at 0, 12, or 24 h after reperfusion. The left kidneys were gathered and rinsed with ice-cold normal saline, followed by dissection and fixation in 10% neutral-buffered formalin or rapid storage in liquid nitrogen for further analysis. The experimental procedures were consistent with the experimental protocols approved by the Bioethics Committee of Renmin Hospital of Wuhan University.

### 2.3. Cell Culture

The human renal proximal tubular epithelial cell line (HK-2) was supplied by the American Type Culture Collection. The HK-2 cells were incubated at 37°C in Dulbecco's Modified Eagle's Medium (DMEM) (Invitrogen, USA), including nonessential amino acids, 0.05 mg/ml bovine pituitary extract, 50 ng/ml human recombinant epidermal growth factor, 100 units/ml penicillin, 100 *μ*g/ml streptomycin, and 10% fetal bovine serum (FBS), under conditions of 5% carbon dioxide (CO_2_) and 95% air. The cell hypoxia/reoxygenation (H/R) model was established as follows. Briefly, HK-2 cells were exposed to hypoxia (37°C, 1% oxygen, 94% nitrogen, and 5% CO_2_) for 12 h in a medium without nutrients (i.e., glucose-free and serum-free) to induce hypoxic injury. Subsequently, the medium was refreshed, and the culture plates were placed in a normal cell incubator (5% CO_2_ and 95% air) for reoxygenation for 2 h, 3 h, or 4 h, according to the experimental design. The control group was cultured in a regular incubator (i.e., 5% CO_2_ and 95% air).

### 2.4. Real-Time Quantitative Reverse Transcription-Polymerase Chain Reaction (qRT-PCR)

RNAiso Plus (TaKaRa Biotech, Dalian, China) was used to extract total RNA from frozen kidney tissues and HK-2 cells according to the instructions provided by the manufacturer. Subsequently, the PrimeScript™ RT Reagent Kit (TaKaRa Biotech) was used for reverse transcription into cDNA. In all PCR experiments, the expression of GAPDH was used as the internal reference. The qRT-PCR analysis was performed using the ABI ViiA7 DX System (Foster City, CA, USA). The qRT-PCR primers for the specific target genes (listed below) were designed and synthesized by TaKaRa Biotech:

GAPDH: 5′-TCAAGAAGGTGGTGAAGCAGG-3′(F), 5′-TCAAAGGTGGAGGAGTGGGT-3′(R); PRMT5: 5′-AGAACCGTCCTCCACCTA-3′(F), 5′-CTCCCAGCACCATCAGTA-3′(R); NLRP3: 5′-TTCGGAGATTGTGGTTGGG-3′(F), 5′-AGGGCGTTGTCACTCAGGT-3′(R); caspase-1: 5′-AAGGACAAACCGAAGGTG-3′(F), 5′-GAAGAGCAGAAAGCGATA-3′(R); ASC: 5′-CAGCACCGGGCTGCGCTTAT-3′(F), 5′-CGCATCTTGCTTGGGTTGG-3′(R); IL-1*β*: 5′-CGAATCTCCGACCACCACTA-3′(F), 5′-AGCCTCGTTATCCCATGTGT-3′(R)

The routine qRT-PCR for NLRP3, ASC, caspase-1, IL-1*β*, and PRMT5 was performed as follows: 94°C for 3 min, followed by 30 cycles (25 cycles for *β*-actin) at 94°C for 30 s, 55°C for 30 s, and 72°C for 1 min.

### 2.5. Western Blotting

The total proteins were extracted from kidney tissues and HK-2 cells using a radioimmunoprecipitation assay buffer containing protease inhibitors (Beyotime, Jiangsu, China). Subsequently, a protein assay kit (Bio-Rad, Hercules, CA, USA) was used for quantification, in accordance with the protocol provided by the manufacturer. Electrophoresis on 10% sodium dodecyl sulfate-polyacrylamide gels was performed to separate equal amounts of protein (40 *μ*g/lane), which were rapidly transferred to polyvinylidene fluoride membranes. The membranes were blocked using 5% fat-free milk for 2 h at room temperature. Proteins were incubated with the primary antibodies at the following dilutions: anti-GAPAH (1 : 1,000), PRMT5 (1 : 1,000), Nrf2 (1 : 1,000), HO-1 (1 : 1,000), NLRP3 (1 : 1,000), ASC (1 : 1,000), IL-1*β* (1 : 800), caspase-1 (1 : 2,000), caspase-11 (1 : 1000), and GSDMD-N (1 : 1000). Tris-buffered saline and Tween 20 buffer was used to remove excessive primary antibodies. Subsequently, the membranes were incubated with an appropriate secondary antibody at 37°C for 2 h, followed by removal of excessive secondary antibody and detection of color exposure. The levels of proteins were analyzed using Image Software (NIH, USA).

### 2.6. Renal Function

After reperfusion in vivo, blood samples were collected and centrifuged, and the supernatant was collected. Creatinine and urea commercial kits (Nanjing Jiancheng Bioengineering Institute, Nanjing, China) were used to evaluate the levels of urea nitrogen (BUN) and creatinine (Cr) in the serum, according to the instructions provided by the manufacturer.

### 2.7. Histology Staining

Hematoxylin-eosin staining was performed on sections (4 *μ*m thick) from the renal tissue, which had been fixed in 4% paraformaldehyde and embedded in paraffin. Two pathologists who were experienced in nephropathology and blinded to the group assignments determined morphological changes. The degree of renal tubular injury was assessed according to the pathological scores (0–5 points) [33].

### 2.8. Immunohistochemistry

A commercial chemical kit (Polink-1 one-step polymer detection system; ZSGB-BIO, Beijing, China) was used to perform immunohistochemical staining. Sections (4 *μ*m thick) were incubated with anti-PRMT5, anti-caspase-1, and anti-Ki-67 primary antibodies overnight at 4°C, followed by incubation with secondary antibody at 37°C for 30 min and addition of a coloring agent. Under the microscope, five different fields (×400) were randomly selected to evaluate the intensity of staining. The ratio of optical density, calculated by the software ipwin32 (USA), was used to assess the intensity of staining.

### 2.9. Small Interfering RNA (siRNA) Transfection

SiRNAs specific to PRMT5 were used to transfect HK-2 cells for 6 h. Meanwhile, nontargeting siRNAs (Santa Cruz Biotechnology, Santa Cruz, CA, USA) were employed to transfect HK-2 cells for 48 h as a negative control. The concentration of all transfected siRNAs was 100 *μ*M. Following transfection, the cells were cultured in DMEM/F12 including 0.2% FBS for 48 h. Subsequently, qRT-PCR was used to ascertain the effects of siRNA transfection.

### 2.10. Cell Viability Assay

The Cell Counting Kit-8 (CCK-8) Assay Kit (Jiancheng, Nanjing, China) was used to assess cell viability. HK-2 cells were incubated in 96-well plates, followed by the addition of 10 *μ*l of CCK-8 reagent per well. The plates were incubated for 3 h in the dark. A PerkinElmer Microplate reader (PerkinElmer Victor 1420, USA) was used to measure the absorbance at 450 nm. The percentage of cell viability was determined as follows: (Treatment group optical density/control group optical density) × 100%.

### 2.11. Measurement of ROS Production

A ROS Assay Kit (Nanjing Jiancheng Bioengineering Institute, Nanjing, China) was used to measure the levels of ROS. HK-2 cells were incubated in a medium with 20 *μ*M DCFH-DA solution for 30 min at 37°C in darkness. Subsequently, the cells were trypsinized and washed with cold phosphate-buffered saline. Flow cytometry (FACSCalibur; BD Biosciences, San Jose, CA, USA) was used to measure the levels of ROS.

### 2.12. Measurement of the Level of MDA and SOD Activity

The radioimmunoprecipitation assay buffer was prepared to lyse the HK-2 cells as previously described [34]. The MDA assay kit and SOD assay kit were used to determine the level of MDA and SOD activity in cell lysates according to the instructions provided by the manufacturer.

### 2.13. Assay for the Production of Hydrogen Peroxide (H_2_O_2_)

The Amplex Red assay was conducted to detect the production of H_2_O_2_ as previously described [35]. Briefly, HK-2 cells, as well as pretreated-HK-2 cells with reagents, were cultured in DMEM//F12 including 0.2% FBS. Subsequently, the production of H_2_O_2_ in HK-2 cells was measured using the Amplex Red assay. For the detection of the level of H_2_O_2_ in kidney tissues, the kidneys were primarily perfused and homogenized as previously described [36]. Amplex Red (100 *μ*M), containing 10 U/ml horseradish peroxidase, was used to detect H_2_O_2_ in the homogenate (incubated for 1 h at 37°C) in accordance with the recommendations of the manufacturer. Subsequently, the fluorescence readings were normalized and assessed.

### 2.14. Statistical Analysis

The statistical analysis was conducted using GraphPad Prism version 5.0 (GraphPad Software, USA). All values are expressed as the mean ± standard errorof the mean. One-way analysis of variance was introduced to analyze differences among experimental groups. A *P* < 0.05 denoted statistical significance.

## 3. Results

### 3.1. The Expression of PRMT5 Was Upregulated after Renal I/R

**Figure 1:**
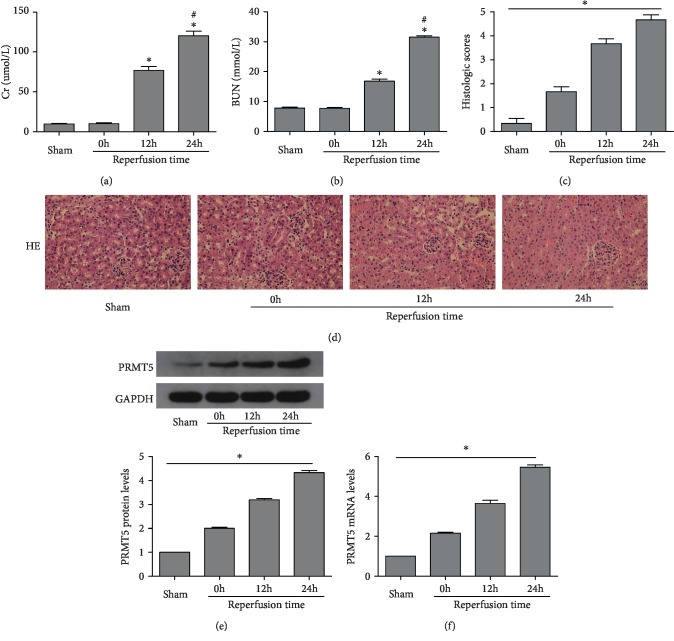
PRMT5 was upregulated and renal function deteriorated after renal ischemia/reperfusion. SCr levels (a) and BUN levels (b) were detected after ischemia and different reperfusion times, 0 h, 12 h, and 24 h. Scores for the histological appearance of acute tubular necrosis (c) and representative images of mouse kidney H-E staining (original magnification ×400) (d). (e) PRMT5 protein levels were detected by western blot analysis after ischemia and different reperfusion time. (f) PRMT5 mRNA levels were detected by real-time RT-PCR after ischemia and different reperfusion time. Values were expressed as the mean ± SEM. ∗*P* < 0.05, relative to the sham group; ^#^*P* < 0.05, relative to the group at reperfusion 12 h, *n* = 6. BUN: blood urea nitrogen; SCr: serum creatinine; H-E: hematoxylin-eosin; I/R: ischemia-reperfusion.

The levels of BUN and Cr were determined, and hematoxylin-eosin staining was performed, to understand the renal function and morphological changes (Figures 1(a)–1(d)). The levels of both BUN and Cr, or the pathological scores of kidney injury, were markedly elevated in the reperfusion group versus the sham group. The expression of PRMT5 was initially determined at 0 h, 12 h, and 24 h after renal I/R using WB and PCR(Figures 1(e) and 1(f)). With the extension of the reperfusion time, the expression of PRMT5 was markedly increased, with the highest expression observed at 24 h versus the sham group. These results suggested that PRMT5 may be involved in the development of kidney damage after I/R, and we performed 24 h reperfusion in the following experiments.

### 3.2. Inhibition of PRMT5 Attenuated Renal Injury and Promoted Tubular Cell Proliferation after I/R

**Figure 2:**
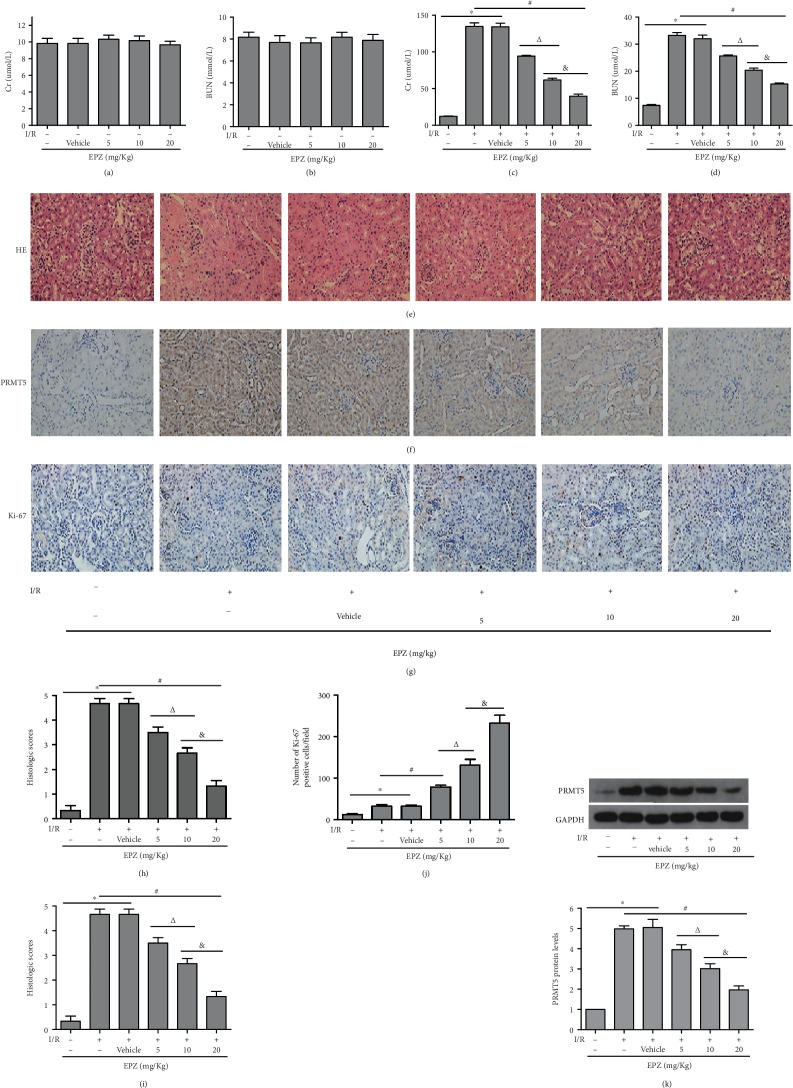
PRMT5 inhibition attenuated renal injury and promoted tubular cell proliferation after I/R. SCr levels (a) and BUN levels (b) were detected after intraperitoneal injection of EPZ with three concentrations (5 mg/kg, 10 mg/kg, and 20 mg/kg) daily for 7 days in normal mice. SCr levels (c) and BUN levels (d) were detected after intraperitoneal injection of EPZ with three concentrations (5 mg/kg, 10 mg/kg, and 20 mg/kg) daily for 7 days before mice undergoing I/R. Scores for the histological appearance of acute tubular necrosis (H) and representative images of mouse kidney H-E staining (original magnification ×400) (e). The representative of immunoblotting of PRMT5 (f) and Ki-67 (g) (original magnification ×400). IOD/area indicated the expression of PRMT5 (i) and the number of Ki-67-positive cells/field indicated the tubular cell proliferation (j). (k) PRMT5 protein levels were detected by western blot analysis. Values were expressed as the mean ± SEM. ^∗^*P* < 0.05, relative to the sham group; ^#^*P* < 0.05, relative to the I/R group; ^Δ^*P* < 0.05, relative to the 5 mg/kg group; ^&^*P* < 0.05, relative to the 10 mg/kg group, *n* = 6. BUN: blood urea nitrogen; SCr: serum creatinine; H-E: hematoxylin-eosin; I/R: ischemia-reperfusion; EPZ: EPZ015666, PRMT5 inhibitor.

The expression of PRMT5 was inhibited by EPZ, an established and powerful inhibitor of PRMT5. Firstly, EPZ (5 mg/kg, 10 mg/kg, or 20 mg/kg daily for 7 days) was administered via intraperitoneal injection in mice, which underwent the sham operation. The evaluation of the level of Cr and BUN showed that EPZ at these three concentrations did not result in marked renal toxicity (Figures 2(a) and 2(b)). I/R induced a significant upregulation of PRMT5 in the operated mice versus the sham group. Notably, this upregulation was inhibited by EPZ. Furthermore, the effect of PRMT5 inhibition was dose-dependent and most pronounced at 20 mg/kg. WB and immunohistochemistry were used to confirm the expression of PRMT5 (Figures 2(f), 2(i), and 2(k)). The levels of Cr and BUN and kidney injury scores were significantly reduced following the administration of EPZ at 20 mg/kg versus I/R group (Figures 2(c)–2(e) and 2(h)). Meanwhile, the expression of Ki-67 indicated that tubular cell proliferation enhanced after the administration of EPZ versus the I/R group (Figures 2(g) and 2(j)). Moreover, the greater the dosage of EPZ, the more obvious the proliferation trend of renal tubular cell was. These results demonstrated that inhibition of PRMT5 markedly attenuated renal injury and promoted tubular cell proliferation after I/R.

### 3.3. Inhibition of PRMT5 Downregulated Oxidative Stress- and Pyroptosis-Related Proteins Induced by Renal I/R

**Figure 3:**
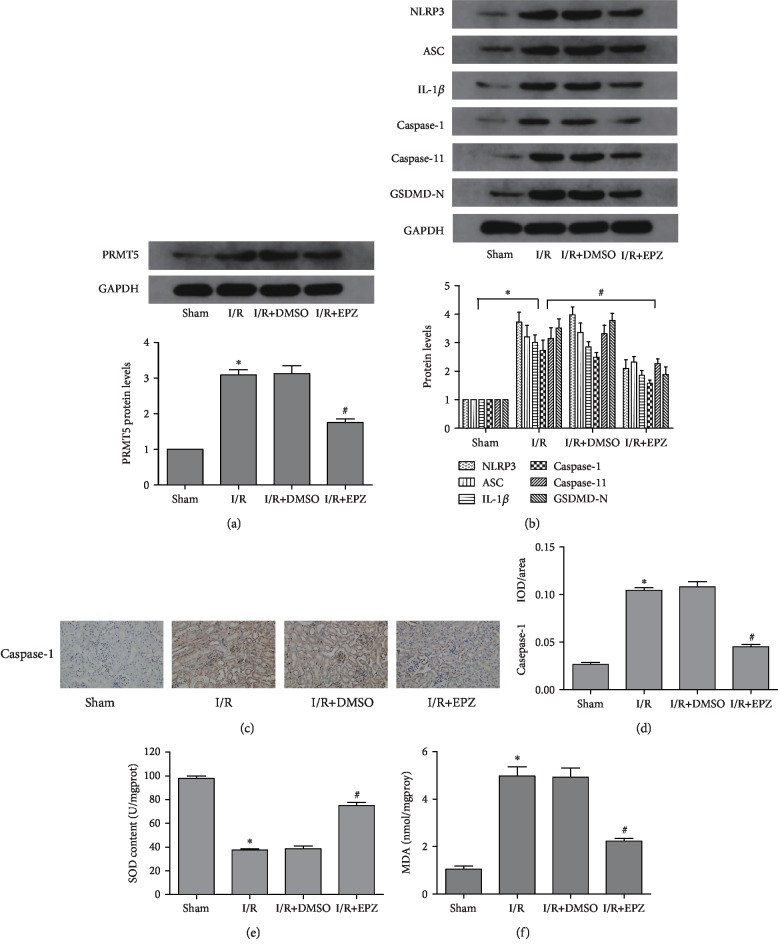
PRMT5 inhibition downregulated oxidative stress and pyroptosisi-related proteins induced by renal I/R. (a) PRMT5 protein levels and (b) pyroptosis-related protein levels were detected by western blot analysis after administration of EPZ. The representative of immunoblotting of caspase-1 (c) (original magnification ×400). IOD/area indicated the expression of caspase-1 (d). (e) SOD activation. (f) MDA contents. Values were expressed as the mean ± SEM. ^∗^*P* < 0.05, relative to the sham group; ^#^*P* < 0.05, relative to the I/R group. *n* = 6. I/R: ischemia-reperfusion; EPZ: EPZ015666, PRMT5 inhibitor; SOD: superoxide dismutase; MDA: malondialdehyde.

Following the administration of EPZ, the expression of PRMT5 was significantly decreased compared with that observed in mice in the I/R groups (Figure 3(a)). In addition, the levels of pyroptosis-related proteins (i.e., NLRP3, ASC, caspase-1, caspase-11, GSDMD-N, and IL-1*β*) were markedly decreased (Figure 3(b)). This trend was observed using WB and immunohistochemistry (Figures 3(c) and 3(d)). Subsequently, we investigated the effects of PRMT5 inhibition on oxidative stress. Similarly, inhibition of PRMT5 drastically reduced the level of MDA and upregulated the activity of SOD compared with those reported in mice in I/R groups (Figures 3(e) and 3(f)). These results verified that inhibition of PRMT5 alleviated oxidative stress and the expression of pyroptosis-related proteins induced by renal I/R.

### 3.4. Pyroptosis Induced by H/R in HK-2 Cells Depends on Oxidative Stress In Vitro

**Figure 4:**
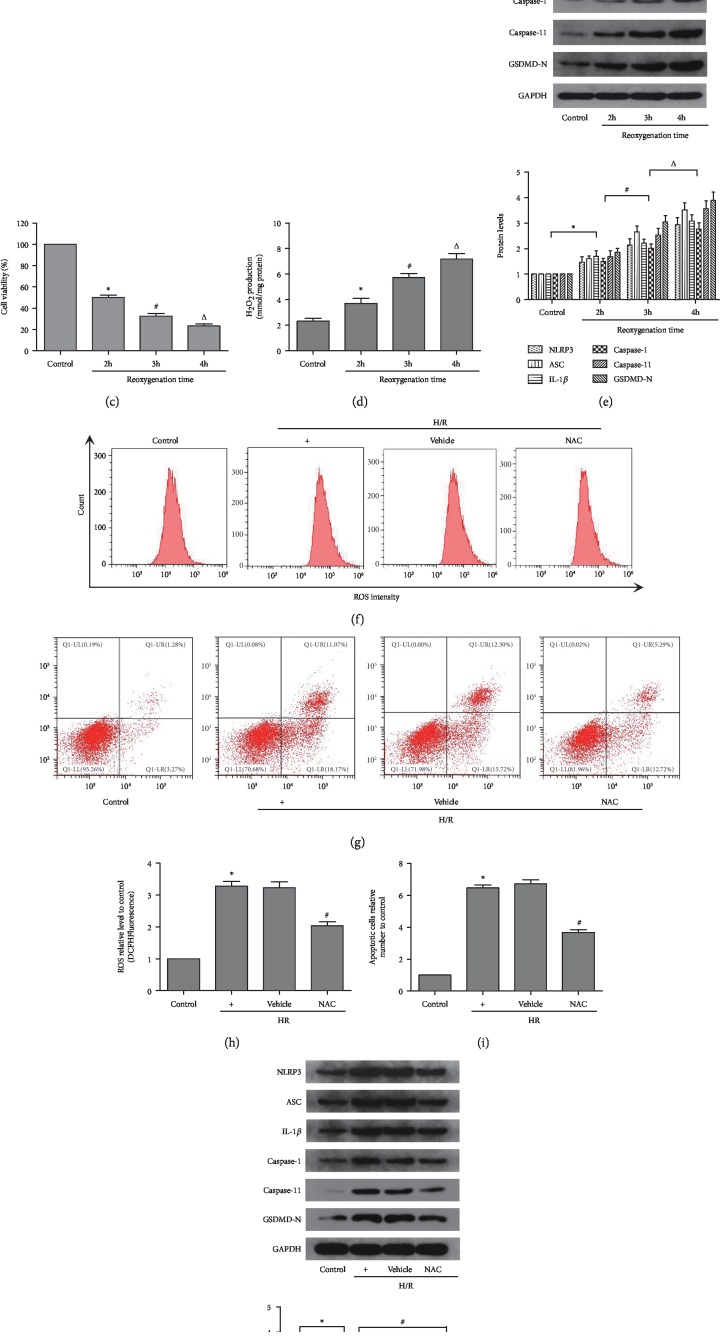
Pyroptosis induce by H/R in HK-2 cells depends on oxidative stress. (a, c) The levels of oxidative stress in different reoxygenation time groups of HK-2 cells was measured by flow cytometry. (b) The cell viability in different reoxygenation time groups of HK-2 cells was measured by CCK-8. (d) An Amplex Red assay showed that hydrogen peroxide was increased with the reoxygenation time extension. (e) Pyroptosis-related protein levels were detected by western blot analysis after different reoxygenation time. (f–i) The apoptosis and the levels of oxidative stress in different intervention groups of HK-2 cells were measured by flow cytometry. (j) Pyroptosisi-related protein levels were detected by western blot analysis after different interventions. Values were expressed as the mean ± SEM. (b–e) ^∗^P < 0.05, relative to the control group; ^#^*P* < 0.05, relative to the reoxygenation time of the 2 h group; ^Δ^*P* < 0.05, relative to the reoxygenation time of the 3 h group, *n* = 6. (h–j) ^∗^*P* < 0.05, relative to the control group; ^#^*P* < 0.05, relative to the H/R group, *n* = 6. H/R: hypoxia/reoxygenation; ROS: reactive oxygen species; NAC: N-acetyl-L-cysteine.

Initially, cell viability was tested using flow cytometry (CCK-8). The results showed that viability of HK-2 cells who underwent H/R declined compared with that reported in the control group (Figure 4(c)). Moreover, with the extension of the reoxygenation time, cell viability was gradually decreased. We examined the production of ROS in HK-2 cells that had undergone hypoxia and subsequent reoxygenation for 2 h, 3, or 4 h using the fluorescent dye DCFH-DA and method for the detection of H_2_O_2_ (Figures 4(a), 4(b), and 4(d)). The analysis showed that H/R caused accumulation of ROS, and specifically H_2_O_2_, in HK-2 cells in a reoxygenation time-dependent manner versus the control group. Meanwhile, the expression of pyroptosis-related proteins (i.e., NLRP3, ASC, caspase-1, caspase-11, GSDMD-N, and IL-1*β*) was notably increased in HK-2 cells in parallel with the extension of reoxygenation time (Figure 4(e)). Subsequently, we investigated the relationship between pyroptosis and oxidative stress. An established antioxidant, NAC (1 *μ*M), was used to reduce the oxidative products and oxidative stress. Following the administration of NAC, the production of ROS, rate of apoptosis, and expression of pyroptosis-related proteins in HK-2 cells were markedly decreased compared with those observed in the H/R groups (Figures 4(f)–4(j)). Therefore, the 4 h reoxygenation time was selected for the subsequent experiment.

### 3.5. Inhibition of PRMT5 Attenuated H/R-Induced Pyroptosis in HK-2 Cells

**Figure 5:**
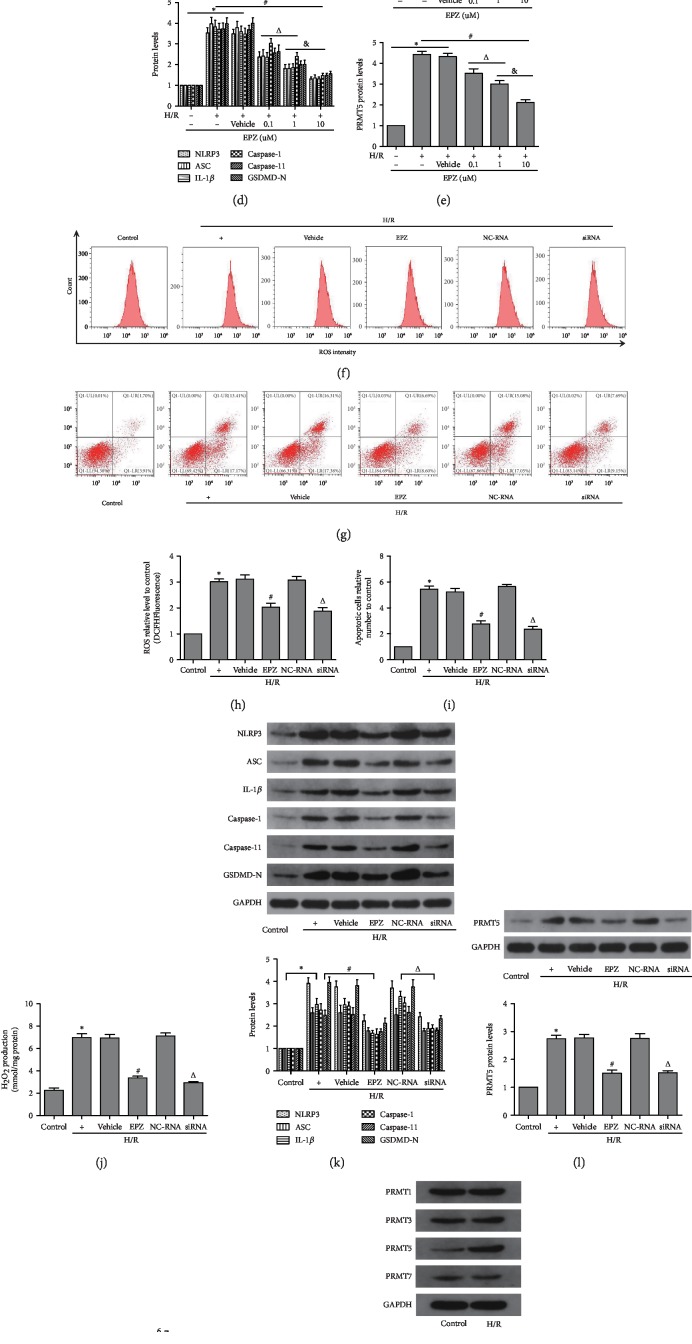
PRMT5 inhibition attenuated H/R-induced pyroptosis in HK-2 cells. (a, b) The cell viability in different EPZ concentration groups of normal HK-2 cells and H/R HK-2 cells was measured by CCK-8, respectively. (c–e) Pyroptosis-related protein levels and PRMT5 protein levels were detected by western blot analysis after application of EPZ with different concentration. (f, h) The levels of oxidative stress in different intervention groups of HK-2 cells was measured by flow cytometry. (g, i) The apoptosis in different intervention groups of HK-2 cells were measured by flow cytometry. (j) An Amplex Red assay was used to show levels of hydrogen peroxide in different intervention groups of HK-2 cells. (k, l) Pyroptosis-related protein levels and PRMT5 protein levels were detected by western blot analysis in different intervention groups of HK-2 cells. (m) Pyroptosis-related protein mRNA levels were detected by real-time RT-PCR in different intervention groups of HK-2 cells. (n) Different subtypes of PRMT were measured by western blot analysis in the H/R group of HK-2 cells. Values were expressed as the mean ± SEM. (a, b, d, and e) ^∗^*P* < 0.05, relative to the control group; ^#^*P* < 0.05, relative to the H/R group; ^Δ^*P* < 0.05, relative to the group with 0.1 uM EPZ. ^&^*P* < 0.05, relative to the group with 1 uM EPZ, *n* = 6. (h–n) ^∗^*P* < 0.05, relative to the control group; ^#^*P* < 0.05, relative to the H/R group; ^Δ^*P* < 0.05, relative to the NC-RNA group, *n* = 6. H/R: hypoxia/reoxygenation; ROS: reactive oxygen species; EPZ: EPZ015666; PRMT5 inhibitor; siRNA: small interfering RNA specific to PRMT5; NC-RNA: nontargeting siRNAs.

Initially, EPZ (0.1 *μ*M, 1 *μ*M, or 10 *μ*M daily for 3 days) was administered to HK-2 cells, which had not undergone H/R. The CCK-8 assay showed that EPZ did not exert a marked cytotoxic effect (Figure 5(a)). Subsequently, EPZ was used in cells that underwent hypoxia for 12 h and reoxygenation for 4 h. Cell viability was prominently decreased in the H/R group versus the control group. Moreover, the increased expression of PRMT5 induced by H/R was notably inhibited by EPZ versus that observed in I/R group. Furthermore, the effect of PRMT5 inhibition was dose-dependent and more pronounced at 10 *μ*M (Figure 5(b)). Additionally, consistent with the decreased expression of PRMT5, the expression of pyroptosis-related proteins was also decreased (Figures 5(c)–5(e)). Therefore, the concentration of 10 *μ*M EPZ was used in the subsequent experiment.

We determined the expression of PRMT1, PRMT3, PRMT5, and PRMT7 in the control and H/R groups (Figure 5(n)). The results demonstrated that only the expression of PRMT5 was significantly different between the control and H/R groups. Subsequently, siRNA against PRMT5 was also used to further verify the effect of PRMT5 inhibition on H/R-induced pyroptosis and oxidative stress in HK-2 cells (Figures 5(f)–5(m)). As shown in the figure, the levels of ROS and rate of apoptosis in cells pretreated with either EPZ or siRNA against PRMT5 were decreased compared with those observed in the H/R and H/R+NC-RNA groups, respectively. Cells were transfected with siRNA against PRMT5, and this transfection exerted a similar effect to that reported with EPZ. We observed that the expression of PRMT5 and pyroptosis-related proteins was markedly decreased compared with that reported in the H/R+NC-RNA group. Similarly, following the application of siRNA against PRMT5 or application of EPZ, the production of ROS and H_2_O_2_ in cells was markedly reduced versus that reported in the H/R+NC-RNA and H/R groups, respectively.

### 3.6. Inhibition of PRMT5 Blocked Oxidative Stress via Activation of NF-E2-Related Factor/Heme Oxygenase-1 (Nrf2/HO-1) in HK-2 Cells

**Figure 6:**
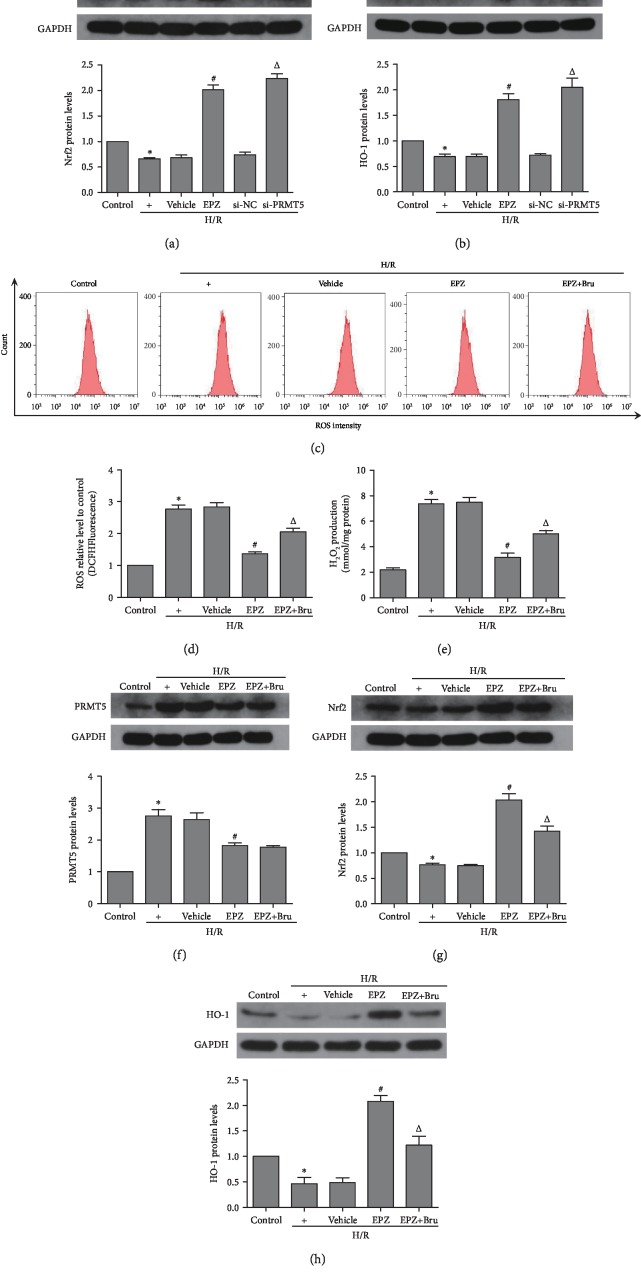
PRMT5 inhibition blocked oxidative stress via activating Nrf2/HO-1 in HK-2 cells. (a, b) Nrf2 protein levels and HO-1 protein levels were detected by western blot analysis in different intervention groups of HK-2 cells. (c, d) The levels of oxidative stress in different intervention groups of HK-2 cells was measured by flow cytometry. (e) An Amplex Red assay was used to show levels of hydrogen peroxide in different intervention groups of HK-2 cells. (f–h) PRMT5 protein levels, Nrf2 protein levels, and HO-1 protein levels were detected by western blot analysis in different intervention groups of HK-2 cells. Values were expressed as the mean ± SEM. (a, b) ^∗^*P* < 0.05, relative to the control group; ^#^*P* < 0.05, relative to the H/R group; ^Δ^*P* < 0.05, relative to the si-NC group, *n* = 6. (d–h) ^∗^P < 0.05, relative to the control group; ^#^*P* < 0.05, relative to the H/R group; ^Δ^*P* < 0.05, relative to the EPZ group, *n* = 6. H/R: hypoxia/reoxygenation; ROS: reactive oxygen species; EPZ: EPZ015666, PRMT5 inhibitor; si-PRMT5: small interfering RNA specific to PRMT5; si-NC: non-targeting siRNAs; Bru: brusatol, inhibitor of Nrf2.

The Nrf2/HO-1 proteins play a crucial role in the regulation of oxidative stress and inflammation. The expression of Nrf2 and HO-1 proteins was significantly decreased in the H/R group versus the control group, and this trend was reversed after application of siRNA against PRMT5 or application of EPZ (Figures 6(a) and 6(b)). Brusatol, an established inhibitor of Nrf2, was used (40 *μ*M, application 2 h prior to hypoxia) to further investigate the relationship between Nrf2/HO-1 and oxidative stress. Following the application of EPZ and brusatol, the levels of ROS and H_2_O_2_ in HK-2 cells were drastically increased compared with those reported in the EPZ-treated group (Figures 6(c)–6(e)). In addition, the expression of the Nrf2/HO-1 proteins was notably decreased compared with that observed in the EPZ-treated group (Figures 6(g) and 6(h)); however, the expression of PRMT5 did not demonstrate an obvious change (Figure 6(f)). This indicates that the Nrf2/HO-1 pathway is a downstream of PRMT5. Collectively, these results revealed that inhibition of PRMT5 blocked oxidative stress via activation of Nrf2/HO-1 in HK-2 cells.

### 3.7. Inhibition of PRMT5 Attenuated Oxidative Stress and Activated Nrf2/HO-1 Signaling In Vivo

**Figure 7:**
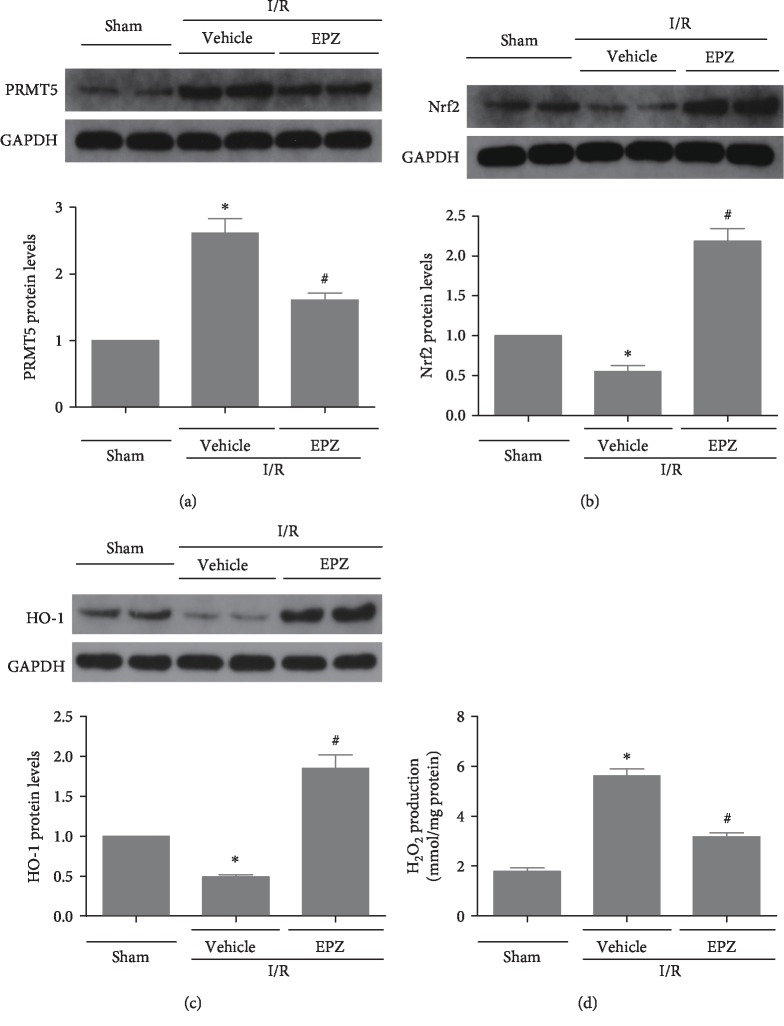
PRMT5 inhibition attenuated oxidative stress and activated Nrf2/HO-1 signaling in vivo. (a–c)PRMT5, Nrf2 and HO-1 protein levels were detected by western blot analysis after administration of the EPZ in the mouse model of renal ischemia reperfusion injury. (d) An Amplex Red assay was used to show levels of hydrogen peroxide after administration of the EPZ in the mouse model of renal ischemia reperfusion injury. Values were expressed as the mean ± SEM. ^∗^*P* < 0.05, relative to the sham groups; ^#^*P* < 0.05, relative to the I/R groups, *n* = 6. I/R: ischemia-reperfusion; EPZ: EPZ015666, PRMT5 inhibitor.

The effects of PRMT5 inhibition on the production of ROS in vitro need to be verified in vivo. As shown in the figure, the increased expression of PRMT5 induced by I/R was repressed by EPZ (Figure 7(a)), and the production of H_2_O_2_ induced by I/R was prominently blocked by the inhibition of PRMT5 (Figure 7(d)). Meanwhile, the Nrf2/HO-1 signal pathway involved in the regulation of ROS production was inhibited by I/R and activated in I/R mice treated with EPZ (Figures 7(b) and 7(c)). In summary, these results indicated that inhibition of PRMT5 may prevent I/R injury through activation of Nrf2/HO-1 signaling and attenuation of ROS-induced inflammation and pyroptosis.

## 4. Discussion

We successfully established the in vivo model of renal I/R injury in mice and the in vitro model of H/R in cells. This study is aimed at investigating the relationship between PRMT5 and renal I/R injury. The results of the present study demonstrated that inhibition of PRMT5 with chemical inhibitor EPZ attenuated I/R-induced pyroptosis gene expression, kidney tissue damage, and renal dysfunction and promoted tubular cell proliferation. The role of PRMT5 was highlighted by Braun et al. who showed that the expression of PRMT5 was upregulated upon I/R insult, and the proliferation of renal tubular epithelium was also increased during injured kidney [10]. However, they failed to further explore the role of PRMT5 in I/R injury through either genetic knockdown or chemical inhibitors of PRMT5. Consistent with a previous study, in the present study, our results showed that PRMT5 expression level was significantly elevated by ischemic injury, and the number of Ki-67-positive nuclei was also increased. However, the number of Ki-67-positive nuclei was further increased after administration of EPZ, indicating that EPZ treatment could promote tubular cell proliferation. In addition, the PRMT5 inhibitor EPZ reduced and even reversed the production of ROS, and this reduction of ROS generation alleviated pyroptosis. Correspondingly, H/R-evoked pyroptosis and renal injury were abolished by either PRMT5 knockdown or treatment with EPZ in vitro. Furthermore, we verified that PRMT5 inhibition blocked ROS-mediated pyroptosis through the Nrf2/HO-1 signal pathway. Hence, the present research demonstrated that PRMT5 may be a promising therapeutic target and EPZ may be a useful agent for the treatment of renal I/R injury.

Increasing evidences suggest that epigenetics, including histone acetylation [37], histone methylation [38], and regulation of microRNA [39], are involved in renal I/R injury. The histone methylation state is controlled by the expression and activity of histone methyltransferases and demethylases, which are important regulators of I/R-induced injury [38, 40]. The lysine or arginine residues of histone are the sites of methylation and demethylation, which are modulated by methyltransferases and demethylases, respectively. Histone methyltransferases contain two primary types, namely, lysine-specific and arginine-specific. PRMTs catalyze arginine monomethylation and asymmetric or symmetric demethylation, which is a conserved and ubiquitous physiological process involved in multiple biological processes [6]. There are two types of PRMTs (i.e., type I and type II). The typical representative of type II PRMTs is PRMT5, which catalyzes the formation of symmetric dimethylarginine [7, 8]. PRMT5 has numerous biological functions [6, 9, 41–43]. Lim et al. showed that ischemia-induced apoptosis in human lung epithelial cells and the lung of miniature pigs may be regulated by the activation of PRMT1 and PRMT5 [9]. In the present study, our findings revealed that PRMT5 participated in renal I/R injury, as evidenced by elevated levels of PRMT5 upon H/R and I/R stimulation in a reperfusion and reoxygenation time-dependent manner, respectively.

Chen et al. reported that inhibition of PRMT5 by EPZ blocked the production of inflammatory factors and inhibited cell proliferation in fibroblast-like synoviocytes from patients with rheumatoid arthritis through the NF-*κ*B and AKT pathways, suggesting that PRMT5 may be a promising target for the prevention of synovial inflammation and destruction [44]. However, the role of PRMT5 in renal I/R-mediated inflammation and the underlying mechanisms had not been previously studied. Of note, inflammation is closely related to pyroptosis [45, 46]. In the present study, we ascertained that treatment with the PRMT5 inhibitor EPZ reduced the expression of pyroptosis-related proteins (i.e., NLRP3, ASC, caspase-1, caspase-11, GSDMD, and IL-1*β*) and increased the expression of Ki-67, and ultimately alleviated renal I/R injury. In addition, by detecting the level of MDA and SOD, we demonstrated that inhibition of PRMT5 reduced the production of ROS after I/R injury.

I/R-induced generation of ROS and the inflammatory response determine the severity of renal I/R injury [47, 48]. I/R-induced ROS results in the release of inflammatory-related signaling factors, such as the NLR inflammasome [49]. The NLRP3 inflammasome, which is composed of NLRP3, ASC, and procaspase-1, is the classic inflammatory complex. It catalyzes the conversion of procaspase-1 to activated caspase-1. Subsequently, the activation of caspase-1 contributes to the maturation and secretion of IL-1*β* [22]. Moreover, the activation of caspase-1 induces pyroptosis, a proinflammatory form of programmed cell death. We performed an in vitro validation in HK-2 cells to verify that H/R-induced pyroptosis relied on oxidative stress.

The findings of the present research supported the notion that the production of ROS in HK-2 cells subjected to H/R injury was augmented compared with the control group. Furthermore, the observed increase in the production of ROS was positively correlated with the longer duration of reoxygenation time. Correspondingly, the expression of NLRP3, ASC, caspase-1, caspase-11, GSDMD-N, and IL-1*β* was increased in parallel with the increased production of ROS. These results are consistent with those reported by numerous previous studies [50, 51]. Interestingly, Kim et al. reported that NLRP3 in renal tubular cells plays a crucial role in mitochondrial ROS production, by binding to the mitochondrial antiviral signal protein after hypoxic injury in an inflammasome-independent manner [52]. The classic antioxidant NAC, which eliminates products of oxidative stress, was used to further investigate the induction of ROS production in relation to the inflammatory response and pyroptosis. The results showed that NAC blocked the production of ROS and inhibited the expression of NLRP3, ASC, caspase-1, caspase-11, GSDMD-N, and IL-1*β* proteins induced by H/R in HK-2 cells, further indicating that H/R-induced pyroptosis remarkably relied on oxidative stress in vitro. A previous study has shown that ischemia/reperfusion could activate caspase-11 and caspase-1 to cleave GSDMD to release GSDMD-N which was associated with the cell plasma membrane to promote leakage of IL-1*β* [53], which was consistent with our findings.

A previous study has reported that the PRMT family is consisted of numerous members involved in various diseases [54]. Our findings illustrated that H/R stimulation hardly affected the levels of other PRMT family members except for PRMT5. These findings encouraged us to focus on the role of PRMT5 in renal I/R injury. The use of EPZ or siRNA against PRMT5 was aimed at limiting the expression of PRMT5 protein or gene, respectively. The results indicated that the production of ROS was decreased by EPZ treatment or PRMT5 knockdown. Simultaneously, the expression of NLRP3, ASC, caspase-1, and IL-1*β* proteins and genes was also downregulated, collectively indicating that inhibition of PRMT5 may attenuate the production of ROS and pyroptosis induced by H/R injury in vitro. These findings were consistent with those reported by previous studies [9, 44]. The enhanced expression and activation of NLRP3, ASC, caspase-1, caspase-11, GSDMD-N, and IL-1*β* are important and typical processes in pyroptosis, which is a proinflammatory form of lytic cell death. Pyroptosis is an important mechanism of host reaction to endogenous injury, and it is initiated by the activation of inflammatory caspases [55]. Compared with other cell death manners, pyroptosis is characterized by inflammatory activation of caspase-1, fragmentation, and nuclear condensation formation of membrane pore, loss of plasma membrane integrity, and extraneous leakage of cytoplasmic content [56]. Notably, it can lead to the release of inflammatory cytokines and tissue injury [28].

Nrf2, which is degraded in the Keap1-dependent pathway, may mediate an array of intracellular antioxidant mechanisms. Under normal conditions, Nrf2 and Keap1 are bound together. Once activated, Nrf2 detaches from the Keap1-Nrf2 bindings and translocates into the nucleus, where it transactivates genes driven by antioxidant response elements (e.g., SOD and HO-1) [27, 57]. HO-1 is thought to be a cytoprotective protein, which is activated by Nrf2. It may protect against apoptosis and oxidative stress under multifarious pathological conditions. Numerous studies have demonstrated that overexpression of HO-1 may alleviate apoptosis and damage following I/R injury [58, 59]. In the present study, we observed that the expression of the Nrf2/HO-1 proteins was markedly increased in EPZ- or siRNA-pretreated HK-2 cells exposed to H/R, which significantly decreased the level of ROS and H_2_O_2_ production. This finding was consistent with those of previous studies [60]. Furthermore, we further investigated the relationship between PRMT5 and Nrf2/HO-1 using brusatol, an established Nrf2 inhibitor. The results showed that brusatol inhibited the expression of Nrf2/HO-1 proteins and increased the levels of ROS and H_2_O_2_. However, it did not affect the expression of PRMT5, indicating that the Nrf2/HO-1 pathway was a downstream of PRMT5. Collectively, these results indicated that inhibition of PRMT5 blocked oxidative stress via activation of Nrf2/HO-1 in HK-2 cells.

We further explored the relationship between PRMT5 and Nrf2 in a mouse model of renal I/R injury. Consistent with the results of the cell experiments, the expression of Nrf2/HO-1 was significantly increased and the level of H_2_O_2_ drastically reduced after EPZ treatment. These results implied that PRMT5 inhibition may attenuate oxidative stress via the activation of Nrf2/HO-1. Khor et al. demonstrated that the activity of Nrf2 was blocked following the methylation of Nrf2 [61]. Although PRMT5 may regulate the expression of Nrf2/HO-1, a limitation of this study is that the epigenetic mechanism of PRMT5 involved in the regulation of Nrf2/HO-1 remains unclear. Unquestionably, the specific interaction between PRMT5 and Nrf2/HO-1 warrants further investigation.

## 5. Conclusions

In conclusion, the present study verified that PRMT5 inhibition offers protection against kidney damage induced by I/R. Furthermore, we demonstrated that PRMT5 inhibition prevented renal cells from ROS-induced inflammation and pyroptosis through the activation of the Nrf2/HO-1 signaling pathway and promoted tubular cell proliferation. Therefore, PRMT5 may be a promising therapeutic target for renal I/R injury.

## Data Availability

The data used to support the findings of this study are available from the corresponding author upon request.
